# Genetic variability in five populations of *Partamona helleri* (Hymenoptera, Apidae) from Minas Gerais State, Brazil

**DOI:** 10.1590/S1415-47572010005000087

**Published:** 2010-12-01

**Authors:** Andreia Arantes Borges, Lucio Antônio de Oliveira Campos, Tânia Maria Fernandes Salomão, Mara Garcia Tavares

**Affiliations:** Departamento de Biologia Geral, Universidade Federal de Viçosa, Viçosa, MGBrazil

**Keywords:** microsatellites, *Partamona helleri*, population genetics, stingless bees

## Abstract

*Partamona* is a Neotropical genus of stingless bees that comprises 33 species distributed from Mexico to southern Brazil. These bees are well-adapted to anthropic environments and build their nests in several substrates. In this study, 66 colonies of *Partamona helleri* from five localities in the Brazilian state of Minas Gerais (São Miguel do Anta, Teixeiras, Porto Firme, Viçosa and Rio Vermelho) were analyzed using nine microsatellite loci in order to assess their genetic variability. Low levels of observed (H_o_ = 0.099-0.137) and expected (H _e_ = 0.128-0.145) heterozygosity were encountered and revealed discrete genetic differentiation among the populations (F _*ST*_ = 0*.*025). AMOVA further showed that most of the total genetic variation (94.24%) in *P. helleri* was explained by the variability within local populations.

*Partamona* is a Neotropical genus of stingless bees that comprises 33 species distributed from Mexico to southern Brazil (Pedro and Camargo, 2003). These bees are found in rain forests, savanna, caatinga and Andean highlands at altitudes greater than 2000 m. Some species are well-adapted to anthropic environments and build their nests in a variety of substrates such as cavities and crevices in walls, among roots of epiphytes, in the bases of palm leaves, in abandoned bird nests, under bridges and in other protected sites; other species nest inside active terrestrial or arboreal termite nests (Camargo and Pedro, 2003).

*Partamona* species build notable nest entrance structures, with special surfaces for incoming/exiting bees. Some of these structures are extremely elaborate and ornamented, and serve as flight orientation targets. The number of individuals per nest ranges from 1000 to 3000 (Michener, 1946). The high abundance of individuals and colonies in several regions has facilitated studies of the genetic variability of populations of these bees. These studies have advanced with the development of molecular techniques such as microsatellites.

To date, microsatellite primers have been developed for eight species of stingless bees: *Melipona bicolor* (Peters *et al.*, 1998), *Scaptotrigona postica* (Paxton *et al.*, 1999), *Trigona carbonaria* (Green *et al.*, 2001), *M. rufiventris* (Lopes *et al.*, 2009), *M. seminigra merrillae* (Francini *et al.*, 2009), *Tetragonisca angustula* (Brito *et al.*, 2009), *Nannotrigona testaceicornis* (Oliveira *et al.*, 2009) and *M. mondury* (Lopes *et al.*, 2010). Many of these primers generated PCR products when used to amplify microsatellite regions in DNA from other species of stingless bees, demonstrating that these markers can be used in populational studies (Paxton *et al.*, 1999; Peters *et al.*, 1999; Paxton, 2000; Cameron *et al.*, 2004; Franck *et al.*, 2004; Carvalho-Zilse and Kerr, 2006; Arias *et al.*, 2006; Francisco *et al.*, 2006; Tavares *et al.*, 2007; Carvalho-Zilse *et al.*, 2009). However, despite the availability of these markers, only two studies have examined the genetic variability of *P. helleri* (Brito, 2005; Francisco *et al.*, 2006).

In the present study, microsatellite markers were used to estimate the number of alleles per locus, the observed heterozygosity and the degree of intra- and inter-populational genetic variation in *P. helleri* (Friese) collected in Viçosa (20°45' S, 42°52' W; 19 colonies), Rio Vermelho (18°17' S, 43°00' W; 12 colonies), Teixeiras (20°39' S, 42°51' W; 12 colonies), Porto Firme (20°40' S, 43°05' W; 11 colonies) and São Miguel do Anta (20°42' S, 42°43' W; 12 colonies) in Minas Gerais state (Figure 1). Twenty adult workers were collected from feral colonies located different distances from each other, in rural and urban areas. The distances between colonies ranged from 5 m (six samples collected from the same wall in a residential area of Viçosa) to 18 km (three colonies collected in a rural area in Rio Vermelho). The remaining 13 colonies collected in Viçosa were 25-1000 m from each other while the distances separating the nine remaining colonies collected in Rio Vermelho ranged from 15 m to 4 km. At Teixeiras and Porto Firme the geographic distance between colonies varied from 85-500 m while colonies from São Miguel do Anta were 150-750 m apart.

The genomic DNA of five adult workers from each colony was extracted according to the protocol recommended by Waldschmidt *et al.* (1997). The amplification reactions consisted of a mixture of 10 μL containing 0.1 mM of each deoxyribonucleoside triphosphate (dATP, dCTP, dGTP, dTTP), 2.5 μM of each *primer*, 0.5 unit of *Taq* polymerase (Phoneutria), 1X buffer containing 15 mM MgCl_2_ and 10 ng of DNA. The *P. helleri* DNA was amplified as reported by Brito (2005) and the amplification products were separated by electrophoresis in 8% non-denaturing polyacrylamide gels and visualized by staining with 0.2% silver nitrate.

The genetic variability in the populations was estimated from the analysis of nine microsatellite loci (Mbi11, 28, 32, 33, 201, 219, 254, 278 and 522) described for *M. bicolor* (Peters *et al.*, 1998), using the following estimates: polymorphic locus percentage (P), mean number of alleles/locus (A_o_) and observed (H_o_) and expected (H_e_) heterozygosities.

The genetic distance between populations was calculated using the Nei (1972) genetic distance as a dissimilarity measurement. The *F* statistic (Wright, 1978) was used to analyze the genetic structure of the populations and analysis of molecular variance (AMOVA; Excoffier *et al.*, 1992) was used to estimate the within and between population genetic variability. All of the statistical analyses were done using the Genes program (Cruz, 2009) with all of the colonies from the same locality being considered as a single population.

Of the nine loci analyzed in *P. helleri*, only two (Mbi254 and Mbi278) were polymorphic (P = 22.22%). Locus Mbi254 generated 5-8 alleles in the different populations while locus Mbi278 generated two alleles in all populations. The number of alleles observed for the Mbi254 locus in *P. helleri* (8) was greater than in *M. bicolor* (3), whereas five alleles were observed for the Mbi278 locus in *M. bicolor* (Peters *et al.*, 1998)*.* The number of alleles identified in *P. helleri* for the loci Mbi28, 201, 278 and 522 was identical to those reported by Francisco *et al.* (2006) for this same species.

Table 1 shows that the observed heterozygosity ranged from 0.099 (Porto Firme) to 0.137 (Rio Vermelho), with a mean of 0.122. The lowest and highest expected heterozygosities were 0.128 and 0.145 for the Viçosa and Rio Vermelho populations, respectively, with a mean of 0.133.

The mean observed heterozygosity for *P. helleri* (H_o_ = 0.122) was much lower than that reported for *M. bicolor* (H_o_ = 0.40) (Peters *et al.*, 1998). Likewise, the mean heterozygosities for populations of *Plebeia remota* (H_o_ = 0.24) (Francisco *et al.*, 2006), *M. rufiventris* (H_o_ = 0.07) and *M. mondury* (H_o_ = 0.12) (Tavares *et al.*, 2007) were also lower than in *M. bicolor*, even when heterologous primers designed specifically for *M. bicolor* were used.

These data confirm that when heterologous primers are used the number of alleles and the heterozygosity values tend to be lower than in the species for which the microsatellite primers were developed (Peters *et al.*, 1998; Paxton *et al.*, 2003; Francisco *et al.*, 2006; Souza *et al.*, 2007). In addition, according to Carvalho-Zilse and Kerr (2006), the facultative polygyny of *M. bicolor* colonies may contribute to the greater genetic variability observed in this species compared to *P. helleri*, the colonies of which are headed by a single queen.

The genetic variability of *Apis mellifera* (Ho = 0.349-0.589) (De La Rúa *et al.*, 2003) was higher than that of *P. helleri*. This difference may be explained by the multiple matings of honeybee queens that have an effective mate number of 12.4 (Estoup *et al.*, 1994). In contrast, stingless bee queens generally mate once (Kerr *et al.*, 1962; Da Silva *et al.*, 1972; Contel and Kerr, 1976; Machado *et al.*, 1984; Peters *et al.*, 1999) and have an effective mate number of 1.06 (Strassmann, 2001). The estimated effective mate number of *Partamona* aff. *cupira*, a species closely related to *P. helleri,* is 0.91 (Peters *et al.*, 1999).

**Figure 1:**
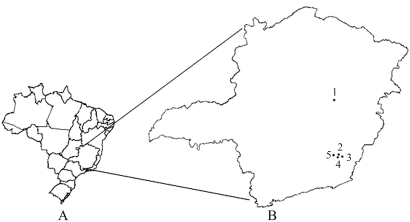
Map of Brazil (A) and Minas Gerais state (B) showing the geographic location of the *P. helleri* populations analyzed: 1 - Rio Vermelho (18°17' S, 43°00' W), 2 - Teixeiras (20°39' S, 42°51' W), 3 - São Miguel do Anta (20°42' S, 42°43' W), 4 - Viçosa (20°45' S, 42°52' W) and 5 - Porto Firme (20°40' S, 43°05' W).

The low level of genetic variability in *P. helleri* may also be explained by this species limited flight distance. The maximum flight distance for *P. cupira* is estimated to be 1159-1710 m (Araújo *et al.*, 2004). Hence, it is reasonable to assume that, after swarming, new *P. helleri* colonies will not disperse more than 1700 m from their maternal colony. In addition, during swarming, the parental colony generally provides the new colonies with food and wax (Kerr, 1987), which further limits their spatial distribution. One consequence of this proximity between parental and daughter colonies is that different alleles may become fixed in adjoining spatial regions. In contrast, honeybees generally swarm over greater distances, with some studies indicating that swarms of *Apis* species may fly 90 km (Otis *et al.*, 1981) to 110 km (Seeley, 1985) from the parental colony. The higher genetic variability of *A. mellifera* compared to *P. helleri* may explain its great adaptability to wide climatic variations and hence its greater ability to exploit different spatial habitats.

The ability of *P. helleri* to adapt to anthropic environments and to use a wide variety of substrates for nesting, together with the fact that many of the sampled colonies were located close to each other, suggests that the populations analyzed were derived from only a few parental colonies (few queens) that encountered favourable conditions for expansion. The establishing of new nests by the dispersing colony in the neighborhood of the parental colony probably led to a decrease in the genetic variability of the population. The artificial transport of *P. helleri* colonies between regions, which could increase the population genetic variability, is not a common practice among beekeepers, which do not rear this species commercially because of its aggressiveness and the low commercial value of its honey.

Nei's genetic distance calculated pairwise between populations varied from 0.00 to 0.0079. These values were consistent with the geographic proximity of the sampled sites (except for Rio Vermelho), and with the fact that most of the alleles were shared by all of the populations. Indeed, the presence of an exclusive allele (allele H of the Mbi254 locus) was detected only in the population from Rio Vermelho, which is at least 270 km away from the other populations. All of the other alleles detected in the two polymorphic loci were present in at least two populations. The presence of this exclusive allele is evidence of restricted genetic flow between this and the other populations of *P. helleri* analyzed.

The presence of an exclusive allele may also indicate the existence of genetic structure among populations. In the present study, genetic structure was initially detected based on differences in the allelic frequencies among populations (data not shown) and further supported by the F_IS_ (Table 1) and mean F_*ST*_ (F_*ST*_ = 0*.*025) values. The F_*ST*_ value indicated that the genetic differentiation among the populations was discrete, whereas AMOVA showed that most (94.24%) of the total genetic variation in *P. helleri* was explained by the genetic diversity within local populations, while 4.76% was distributed among the populations. Based on these findings, we suggest that there are genetic differences among the populations analyzed but that these differences are not great enough to classify the populations as separate subpopulations.

In conclusion, the populations of *P. helleri* analyzed here showed little genetic variability and genetic differentiation. This finding suggests that these populations originated from only a few colonies. However, additional studies are needed to improve our knowledge of the population dynamics and mode of colonization in this stingless bee.

## Figures and Tables

**Table 1:** Estimates of genetic parameters in five *Partamona helleri* populations. N: number of individuals sampled, H_o_: observed heterozygosity, H_e_: expected heterozygosity; F_IS_: fixation index.

Population	N	H_o_	H_e_	F_IS_
São Miguel do Anta	12	0.127	0.129	0.018
Teixeiras	12	0.127	0.129	0.108
Rio Vermelho	12	0.137	0.145	0.128
Porto Firme	11	0.099	0.132	0.112
Viçosa	19	0.119	0.128	0.111

Mean	13	0.122	0.133	

## References

[Araujoetal2004] Araújo E.D., Costa M., Chaud-Neto J., Fowler H.G. (2004). Body size and flight distance in stingless bees (Hymenoptera, Meliponini): Inference of flight range and possible ecological implications. Braz J Biol.

[Ariasetal2006] Arias M.C., Brito R.M., Francisco F.O., Moretto G., Oliveira F.F., Silvestre D., Sheppard W.S. (2006). Molecular markers as a tool for population and evolutionary studies of stingless bees. Apidologie.

[Britoetal2009] Brito R.M., Francisco F.O., Domingues-Yamada A.M.T., Gonçalves P.H.P., Pioker F.C., Soares A.E.E., Arias M.C. (2009). Characterization of microsatellite loci of *Tetragonisca**angustula* (Hymenoptera, Apidae, Meliponini). Conserv Genet Resour.

[CamargoandPedro2003] Camargo J.M.F., Pedro S.R.M. (2003). Meliponini neotropicais: O gênero *Partamona* Schwarz, 1939 (Hymenoptera, Apidae, Apinae). Rev Bras Entomol.

[Cameronetal2004] Cameron E.C., Franck P., Oldroyd P. (2004). Genetic structure of nest aggregations and drone congregations of the southeast Asian stingless bee *Trigona collina*. Mol Ecol.

[Carvalho-ZilseandKerr2006] Carvalho-Zilse G.A., Kerr W.E. (2006). Utilização de marcadores microssatélites para estudos populacionais em *Melipona scutellaris* (Apidae, Meliponini). Magistra.

[Carvalho-Zilseetal2009] Carvalho-Zilse G.A., Costa-Pinto M.F.F., Nunes-Silva C.G., Kerr W.E. (2009). Does beekeeping reduce genetic variability in *Melipona scutellaris* (Apidae, Meliponini)?. Genet Mol Res.

[ContelandKerr1976] Contel E.P.B., Kerr W.E. (1976). Origin of males in *Melipona subnitida* estimated from data of an isozymic polymorphic system. Genetica.

[DaSilvaetal1972] Da Silva D.L.N., Zucchi R., Kerr W.E. (1972). Biological and behavioral aspects of the reproduction in some species of *Melipona* (Hymenoptera, Apidae, Meliponinae). Anim Behav.

[DeLaRuaetal2003] De La Rúa P., Galián J., Serrano J., Moritz F.A. (2003). Genetic structure of Balearic honeybee populations based on microsatellite polymorphism. Genet Sel Evol.

[Estoupetal1994] Estoup A., Solignac M., Cornuet J.M. (1994). Precise assessment of the number of patrilines and of genetic relatedness in honeybee colonies. Proc R Soc Lond B.

[Excoffieretal1992] Excoffier L., Smouse P.E., Quattro J.M. (1992). Analysis of molecular variance inferred from metric distances among DNA haplotypes: Application to human mitochondrial DNA restriction data. Genetics.

[Francinietal2009] Francini I.B., Sforça D.A., Sousa A.C.B., Campos T., Cidade F.W., Zucchi M.I., Souza A.P., Nunes-Silva C.G., Carvalho-Zilse G.A. (2009). Microsatellite loci for an endemic stingless bee *Melipona seminigra merrilae* (Apidae, Meliponini) from Amazon. Conserv Genet Resour.

[Franciscoetal2006] Francisco F.O., Brito R.M., Arias M.C. (2006). Allele number and heterozygosity for microsatellite loci in different stingless bee species (Hymenoptera, Apidae, Meliponini). Neotrop Entomol.

[Francketal2004] Franck P., Cameron E., Good G. (2004). Nest architecture and genetic differentiation in a species complex of Australian stingless bees. Mol Ecol.

[Greenetal2001] Green C.L., Franck P., Oldroyd P. (2001). Characterization of microsatellite loci for *Trigona carbonaria*, a stingless bee endemic to Australia. Mol Ecol.

[Kerr1987] Kerr W.E. (1987). Abelhas indígenas brasileiras (meliponíneos) na polinização e na produção de mel, pólen, geoprópolis e cera. Inf Agropec.

[Kerretal1962] Kerr W.E., Zucchi R., Nakadaira J.T., Butolo J.E. (1962). Reproduction in the social bees (Hymenoptera, Apidae). J N Y Entomol Soc.

[Lopesetal2009] Lopes D.M., Silva F.O., Salomão T.M.F., Campos L.A.O., Tavares M.G. (2009). Microsatellite loci for the stingless bee *Melipona rufiventris* (Hymenoptera, Apidae). Mol Ecol Resour.

[Lopesetal2010] Lopes D.M., Silva F.O., Salomão T.M.F., Campos L.A.O., Tavares M.G. (2010). A scientific note on the characterization of microsatellite loci for *Melipona mondury* (Hymenoptera, Apidae). Apidologie.

[Machadoetal1984] Machado M.F.P.S., Contel E.P.B., Kerr W.E. (1984). Proportion of males sons-of-the-queen and sons-of-workers in *Plebeia droryana* (Hymenoptera, Apidae) estimated from data of an MDH isozymic polymorphic system. Genetica.

[Michener1946] Michener C.D. (1946). Notes on the habits of some Panamanian stingless bees (Hymenoptera, Apidae). J N Y Entomol Soc.

[Nei1972] Nei M. (1972). Genetic distance between populations. Am Nat.

[Oliveiraetal2009] Oliveira E.J.F., Freitas G.S., Fonseca A.S., Sousa A.C.B., Campos T., Assis A.F., Souza A.P., Contel E.P.B., Soares A.E.E. (2009). Isolation and characterization of microsatellite markers from the stingless bee *Nannotrigona testaceicornis*. Conserv Genet Resour.

[Otisetal1981] Otis G.W., Winston M.L., Taylor O.R. (1981). Engorgement and dispersal of Africanized honeybee swarms. J Apic Res.

[Paxton2000] Paxton R.J. (2000). Genetic structure of colonies and a male aggregation in the stingless bee *Scaptotrigona postica*, as revealed by microsatellites analysis. Insectes Soc.

[Paxtonetal1999] Paxton R.J., Wei[##946 ]schuh N., Engels W., Quezada-Euán J.J.G. (1999). Characterization of dinucleotide microsatellite locos for stingless bees. Mol Ecol.

[Paxtonetal2003] Paxton R.J., Bego L.R., Shah M.M. (2003). Low mating frequency of queens in the stingless bee *Scaptotrigona postica* and worker maternity of males. Behav Ecol Sociobiol.

[PedroandCamargo2003] Pedro S.R.M., Camargo J.M.F. (2003). Meliponini Neotropicais: O gênero *Partamona* Schwarz, 1939 (Hymenoptera, Apidae). Rev Bras Entomol.

[Petersetal1998] Peters J.M., Queller D.C., Imperatriz-Fonseca V.L., Strassmann J.E. (1998). Microsatellite loci for stingless bees. Mol Ecol.

[Petersetal1999] Peters J.M., Queller D.C., Imperatriz-Fonseca V.L., Roubik D.W., Strassmann J.E. (1999). Mate number, kin selection and social conflicts in stingless bees and honeybees. Proc R Soc Lond B.

[Seeley1985] Seeley T.D. (1985). Honeybee Ecology: A Study of Adaptation in Social Life.

[Souzaetal2007] Souza R.O., Cervini M., Del Lama M.A., Paxton R.J. (2007). Microsatellite loci for euglossine bees (Hymenoptera, Apidae). Mol Ecol Notes.

[Strassmann2001] Strassmann J. (2001). The rarity of multiple mating by females in the social Hymenoptera. Insectes Soc.

[Tavaresetal2007] Tavares M.G., Dias L.A.S., Borges A.A., Lopes D.M., Busse A.H.P., Costa R.G., Salomão T.M.F., Campos L.A.O. (2007). Genetic divergence between populations of the stingless bee uruçu amarela (*Melipona rufiventris* group, Hymenoptera, Meliponini): Is there a new *Melipona* species in the Brazilian state of Minas Gerais?. Genet Mol Biol.

[Waldschmidtetal1997] Waldschmidt A.M., Salomão T.M.F., Barros E.G., Campos L.A.O. (1997). Extraction of genomic DNA from *Melipona quadrifasciata* (Hymenoptera, Apidae, Meliponinae). Braz J Genet.

[Wright1978] Wright S. (1978). Evolution and the Genetics of Populations. Variability Within and Among Natural Populations.

